# The application of artificial neural networks in modeling and predicting the effects of melatonin on morphological responses of citrus to drought stress

**DOI:** 10.1371/journal.pone.0240427

**Published:** 2020-10-14

**Authors:** Marziyeh Jafari, Alireza Shahsavar

**Affiliations:** Department of Horticultural Science, College of Agriculture, Shiraz University, Shiraz, Iran; South China University of Technology, CHINA

## Abstract

Drought stress as one of the most devastating abiotic stresses affects agricultural and horticultural productivity in many parts of the world. The application of melatonin can be considered as a promising approach for alleviating the negative impact of drought stress. Modeling of morphological responses to drought stress can be helpful to predict the optimal condition for improving plant productivity. The objective of the current study is modeling and predicting morphological responses (leaf length, number of leaves/plants, crown diameter, plant height, and internode length) of citrus to drought stress, based on four input variables including melatonin concentrations, days after applying treatments, citrus species, and level of drought stress, using different Artificial Neural Networks (ANNs) including Generalized Regression Neural Network (GRNN), Radial basis function (RBF), and Multilayer Perceptron (MLP). The results indicated a higher accuracy of GRNN as compared to RBF and MLP. The great accordance between the experimental and predicted data of morphological responses for both training and testing processes support the excellent efficiency of developed GRNN models. Also, GRNN was connected to Non-dominated Sorting Genetic Algorithm-II (NSGA-II) to optimize input variables for obtaining the best morphological responses. Generally, the validation experiment showed that ANN-NSGA-II can be considered as a promising and reliable computational tool for studying and predicting plant morphological and physiological responses to drought stress.

## Introduction

It is well documented that drought significantly affects agricultural and horticultural productivity in many parts of the world [1]. Indeed, drought and heat stresses as a direct effect of global climate change lead to the disruptions in morphological, physiological, biochemical, and molecular responses of the plants [2, 3]. Therefore, plant growth and development, as well as agricultural productivity, are remarkably influenced by water scarcity as a major environmental restriction [4]. Drought stress also decreases canopy size, photosynthetic potential due to the acceleration of the leave senescence and chlorophyll degradation which ultimately leads to the lower crop yield [5–7]. In recent years, genetic engineering methods have been suggested as promising approaches for improving plant tolerance to different stresses in particular drought stress. However, genetic manipulation (GM) methods are unacceptable in many parts of the world due to GM product restriction and can be also considered as complicated, time-consuming, and expensive methods [8]. Nowadays, the application of inexpensive, economic, efficient, and promising compounds such as melatonin have been significantly attracted many plant biologists for improving plant tolerance to different abiotic and biotic stresses. Melatonin is categorized as a novel plant growth regulator (PGR) which exists in various levels in different plant cells and tissues [9, 10]. Melatonin has a direct function in alleviating various stresses via scavenging both reactive nitrogen species (RNS) and reactive oxygen species (ROS) and plays also an indirect role in improving the photosynthesis, recovering leaf ultrastructure, increasing antioxidant activities, and stimulating and regulating plant growth regulators in plants [10, 11]. Therefore, it is necessary to apply exogenous melatonin for coping adverse environmental conditions because, sometimes, the endogenous level of melatonin is insufficient for this aim. Several studies have shown that the application of exogenous melatonin could significantly improve stress tolerance in different plants such as tobacco [12], *Camellia sinensis* [13], cucumber [14], soybean [15], alfalfa [16], rice [17], maize [18], cotton [19], grape [20], and kiwi [21]. Therefore, applying melatonin can be considered as a short-term and promising approach for alleviating drought stress in various economically important plants such as citrus. Citrus plants are widely cultivated in different tropical and subtropical parts of the world, where water scarcity is a main environmental limiting factor in agricultural productivity [22]. Decreasing in leaf area and photosynthetic potential as well as increasing in root length are the most common morphological responses of citrus to drought stress [10, 11]. However, the effect of melatonin on the plant morphological and physiological responses to drought stress can be considered as a multivariable process and viewed by different factors such as genotype, environmental conditions, and the concentration of melatonin, and the level of water scarcity. These factors lead to categorize this process as a complex and non-linear biological process. Thus, traditional statistical and computational methods such as simple regression cannot be considered as an appropriate approach for studying the effect of melatonin on plant biological responses to drought stress [23]. Hence, there is a serious need to use nonlinear statistical methodology such as artificial neural networks (ANNs). The reliability and accuracy of different ANNs such as Radial basis function (RBF), Multilayer Perceptron (MLP), and Generalized Regression Neural Network (GRNN) have been previously proven in different fields of science and technology such as *in vitro* culture, prediction of microRNAs and transcription factors (TFs), analysis of plant promoters, remote sensing studies, genome prediction, and phenomics studies [24–27]. ANNs are a type of nonlinear computational methods, which is applied for different aims such as clustering, predicting, and classifying the complex systems [28]. ANNs are able to identify the relationship between output and input variables and recognize the inherent knowledge existent in the datasets without previous physical considerations [29]. However, ANNs do not present a neat mathematical formula that illustrates the relative relationship of each independent variable in the model. Hence, ANNs are considered as a “black box”. ANNs consist of numerous highly interconnected processing neurons that work in parallel to find a solution for a particular problem. ANNs are learned by example. The examples should be carefully chosen otherwise time is wasted or even worse the model might be working inaccurately [23]. However, difficulty in achieving an optimized solution can be considered as one of the demerit points of most machine learning algorithms [23, 30, 31]. To overcome this bottleneck, Zhang *et al*. [32] employed the genetic algorithm (GA) as one of the common optimization algorithms for optimizing relative humidity, light duration, agar concentration, and culture temperature in order to maximize indirect shoot organogenesis in *Cucumis melo*. In another study, Non-dominated Sorting Genetic Algorithm-II (NSGA-II) was employed to optimize different types and concentrations of disinfectants as well as immersion time for maximizing explant viability and minimizing in vitro contamination in chrysanthemum [33]. However, most studies have found the optimized solution by trials and error [23, 34, 35]. NSGA-II is the search algorithms inspired by natural selection and genetics concepts. The fundamental principles of NSGA-II are the creation of an initial population of search solutions and then elite search solutions were selected for crossover using a roulette wheel selection method, which will ultimately be the best solution among them [36]. Therefore, this study has attempted to apply the NSGA-II to find the optimal levels of different factors involved in morphological responses to drought stress.

Considering the dominance of water scarcity in the south of Iran, which can affect the yield, growth, development, and quality of citrus fruits in these regions, the current study was aimed to investigate the effect of foliar melatonin on morphological parameters under different levels of water scarcity in Mexican lime (*Citrus aurantifolia* Swingle) and Persian lime (*Citrus latifolia* Tanaka) as the most commercially important Citrus plants by using ANNs. Three ANNs including, MLP, RBF, and GRNN were employed to compare their prediction accuracy and introduce the appropriate ANN for modeling and predicting morphological parameters under drought stress. Also, GA was linked to the best model to find the optimal condition for achieving the best morphological parameters. According to the best of our knowledge, this investigation is the first comparing ANNs study in the field of stress physiology.

## Material and method

### Plant material

This study was carried out at the greenhouse of College of Agriculture, Shiraz University, Iran, in September 2019 based on a completely randomized design in a factorial arrangement with 3 factors, including citrus species (Mexican lime and Persian lime), melatonin concentrations (0, 50, 100 and 150 μM), and level of water content (100, 75 and 40% of field capacity (FC)), and four replications. One-year-old seedlings of two citrus species, Mexican lime (*Citrus aurantifolia* Swingle) and Persian lime (*Citrus latifolia* Tanaka), were prepared from a commercial nursery (Jahrom, Iran). Both species were planted in plastic pots containing soil + leaf litter (3:2 w/w) and kept in the greenhouse under natural photoperiod at 25±2°C and 80% relative humidity and irrigated three times a week with 1/2 strength Hoagland solution. Melatonin treatments were implemented three times per week for 2 months by using a manual pump (30 ml per plant). The pots were also weighed every two days for applying drought stress (40, 75, and 100% FC). The morphological parameters including leaf length (cm), number of leaves/plants, crown diameter, plant height (cm), and internode length (cm) were measured after 15, 30, 45, and 60 days of the planting.

### Modeling procedures

The obtained data were used for further analysis by using three types of ANNs including MLP, RBF, and GRNN in order to predict and model the morphological responses to melatonin under drought stress. Before modeling, to achieve the minimum mean squared error (MSE), the datasets were normalized between -1 and 1.

The input variables were melatonin concentrations, days after applying treatments, citrus species, and level of drought stress. Also, morphological responses including leaf length, number of leaves/plants, crown diameter, plant height, and internode length were chosen as target variables. To train and test each model, 70 and 30% of the data lines were randomly selected, respectively.

### MLP model

The MLP as one of the most well-known ANNs includes one or more hidden layers, an input layer, and an output layer. A supervised training procedure is implemented by MLP that provides input and output variables to the network; the training set continues until the following equation would be minimized:

$$E=\frac{1}{K}\sum_{k=1}^{K}{\left(y_{k}-{\hat{y}}_{k}\right)}^{2}$$ (1)

where *K*, *y*_*k*_, and ${\hat{y}}_{k}$ are the number of datapoints, the *k*^*th*^ observed data, and the *k*^*th*^ forecasted data, respectively. In a three-layer MLP with *n* inputs and *m* neurons in the hidden layer $\hat{y}$ determined as:

$$\hat{y}=f[\sum_{j=1}^{m}w_{j}.g(\sum_{i=1}^{n}w_{ji}x_{i}+w_{j0})+w_{o}]$$ (2)

where *x*_*i*_ is the *i*^*th*^ input variable, *w*_*0*_ represents bias related to the neuron of output, *w*_*j0*_ is bias of the *j*^*th*^ neuron of hidden layer, *f* represents transfer functions for the output layer, *g* is the transfer functions for hidden layer, *w*_*ji*_ is the weight connecting the *j*^*th*^ neuron of hidden layer and the *i*^*th*^ input variable, and *w*_*j*_ represents weight linking the neuron of output layer and the *j*^*th*^ neuron of hidden layer.

Determining the construction of the MLP has the main function in its performance [37, 38]. In the construction of this model, it is necessary to determine the number of neurons in each layer and the number of hidden layers [38]. Hornik *et al*. [39] revealed that the MLP with a sigmoid transfer function is general approximators; which shows that it could be trained to build any construction between the input and output variables. Therefore, the number of neurons in the hidden layer plays an important role in determining the construction of the MLP. Some studies [25] have recommended the proper number of neurons (*m*) based on the number of data (*K*) or the number of input (*n*). For example, Tang and Fishwick [40], Wong [41], and Wanas *et al*., [42] suggested "n", "2n", and "log (K)" as the suitable neuron number. Eventually, the optimal number of neurons in the hidden layer should be calculated by using trial and error, however, the reported offers could be employed as an initiating point. A large number of neurons contributes to the complexity of the network while a low number of them makes for simplicity of the network, therefore should be noted that a too simple network results in under-fitting, and conversely, becoming too complex causes over-fitting [37, 43].

### RBF model

RBF is a three-layer ANN consisting of an input layer, a hidden layer, and an output layer. This is the basis and principal for radial basis networks, which organizes statistical ANNs. Statistical ANNs refer to networks which in contrast to the traditional ANNs implement regression-based approaches and have not been emulated by the biological neural networks [44]. In an RBF model, Euclidean distance between the center of each neuron and the input is considered as an input of transfer function for that neuron. The most well-known transfer function in RBF is the Gaussian function, which is determined based on the following equation:

$$f(X_{r},X_{b})=e^{-{\left[∥X_{r}-X_{b}∥*0.8326/h\right]}^{2}}$$ (3)

where *X*_*r*_, *X*_*b*_, and *h* are input with unknown output, observed inputs in time *b*, and spread, respectively. The output of the function close to 1 when ∥*X*_*r*_ − *X*_*b*_∥ approaches 0 and close to 0 when ∥*X*_*r*_ − *X*_*b*_∥ approaches a large value. Finally, dependent variable (*Yr*) by predictor *X*_*r*_ is determined as follows:

$$Y_{r}=\sum_{b=1}^{m}w_{b}*f(X_{r},X_{b})+w_{0}$$ (4)

where *w*_*0*_ and *w*_*j*_ are the bias and weight of linkage between the *b*^th^ hidden layer and the output layer, respectively.

### GRNN model

GRNN introduced by Specht [45] is another kind of statistical ANNs with a very fast training process. In GRNN model, the number of observed data and the number of neurons in the hidden layer are equal. This model consists of an input layer, pattern layer, summation layer, and output layer. The pattern layer is completely connected to the input layer. D-summation and S-summation neurons of the summation layer are connected to the output derived from each neuron of the pattern layer. D-summation and S-summation neurons calculate the sum of the unweighted and weighted of the pattern layer, respectively. The connection weight between S-summation neuron and a neuron of the pattern layer is equal to the target output, while the connection weight for D-summation is unity. The output layer obtains the unknown value of output corresponding to the input vector, only via dividing the output of each S-summation neuron through the output of each D-summation neuron [46]. Consequently, the following equation is used to determine the output value:

$$Y_{r}=\frac{\sum_{b=1}^{m}T_{b}.f(X_{r},X_{b})}{\sum_{b=1}^{m}f(X_{r},X_{b})}$$ (5)

where *Y*_*r*_ represents the output value, and *T*_*b*_ is target associated with the *b*^*th*^ observed data.

### Performance measures

To assess and compare the accuracy of mentioned models, three following performance measures including *R*^*2*^ (coefficient of determination), Root Mean Square Error (RMSE), and Mean Bias Error (MBE) were used:

$$R^{2}={\left[\frac{\sum_{t=1}^{T}(y_{t}-\bar{y})(\hat{y_{t}}-\hat{\bar{y}})}{\sqrt{\sum_{t=1}^{T}(y_{t}-\bar{y})}\;\sqrt{\sum_{t=1}^{T}(\hat{y_{t}}-\hat{\bar{y}})}}\right]}^{2}$$ (6)

$$MBE=1/n\sum_{i=1}^{n}\left(y_{i}-{\hat{y}}_{i}\right)$$ (7)

$$RMSE=\sqrt{(\sum_{i=1}^{n}{\left(y_{i}-{\hat{y}}_{i}\right)}^{2})/n}$$ (8)

Where *y*_*t*_ $\overset{-}{y}$, $\hat{y_{t}}$, and *T* are the *t*^th^ observed data, the mean of observed values, the mean of predicted values, and total number of predicted values, respectively. Greater R^2^ and smaller RMSE and MBE indicated better performance of the constructed models [47–49].

### Optimization process by Non-dominated Sorting Genetic Algorithm-II (NSGA-II)

NSGA-II was implemented to optimize the value of input variables in order to find the best morphological responses. Also, a roulette wheel selection method was applied to choose the elite population for crossover. To obtain the best fitness, the initial population, generation number, mutation rate, and crossover rate were respectively adjusted to 200, 1000, 0.5, and 0.7. In the current study, the ideal point of Pareto was selected such that studied parameters became the maximum.

### Sensitivity analysis

Sensitivity analysis was conducted to identify the importance degree of input variables (melatonin concentrations, citrus species, days after applying treatments, and level of drought stress) on the target variables (leaf length, number of leaves/plants, crown diameter, plant height, and internode length). The sensitivity of these parameters was measured by the criteria including variable sensitivity error (VSE) value displaying the performance (root mean square error (RMSE)) of GRNN-GA model when that input variable is removed from the model. Variable sensitivity ratio (VSR) value was determined as ratio of VSE and GRNN-NSGA-II model error (RMSE value) when all input variables are available. A higher important variable in the model was detected by higher VSR.

MATLAB (Matlab, 2010) software was employed to write codes and run the model.

### Validation experiments

The melatonin concentrations, days after applying treatments, citrus species, and level of drought stress optimized by NSGA-II were experimentally tested to evaluate the efficiency of the GRNN-NSGA-II model.

## Result

### The effects of exogenous melatonin on plant growth and evaluation of drought tolerance

Based on our results, in some parameter there were no remarkable differences among melatonin-treated and non-treated plantlets under the well-irrigated (control) condition. On the other hand, drought stress had adverse impacts on morphological traits. When irrigation was stopped and progressive drought stress treatments were implemented, the morphological parameters significantly changed; however, melatonin-treated plantlets exhibited greener leaf tissues than the non-treated seedlings. Also, the highest length of seedlings was observed in plantlets treated by 100 μM melatonin which was significantly longer than control plantlets (non-treated seedlings). Moreover, the plantlets under drought stress displayed a significant decrease in crown diameter and leaf length (Table 1). The leaf elongation of both genotypes was significantly promoted by application melatonin. Moreover, the maximum crown diameter as well as leaf and internode length were obtained through the application of 50 and 100 μM melatonin (Table 1). Also, a relative decrease in plant height and crown diameter was observed in 150 μM melatonin treatment, indicating the inhibitory impact of higher doses of melatonin (Table 1). Generally, most morphological traits (leaf number and length, internode length, shoot height, and crown diameter) were significantly declined under drought stress. On the other hand, our results showed that the exogenous application of appropriate concentration of melatonin can help the genotypes resist the negative impacts of drought stress.

**Table 1:** Effect of melatonin concentrations, days after applying treatments, citrus species, and level of drought stress on morphological responses including leaf length, number of leaves/plants, crown diameter, plant height, and internode length.

Melatonin concentration (μM)	Level of drought stress (% of field capacity)	Days after applying treatments	Citrus species (lime)	Number of leaves/plants	Leaf length (cm)	Internode length (cm)	Crown diameter (cm)	Plant height (cm)
0	100	15	Persian	678.00	12.675	5.390	3.450	178
0	100	30	Persian	664.25	12.925	5.765	3.447	178.7
0	100	45	Persian	651.50	13.075	6.022	3.435	180.1
0	100	60	Persian	644.75	13.075	6.197	2.130	180.5
0	75	15	Persian	168.25	3.650	1.375	1.180	110.3
0	75	30	Persian	162.75	3.375	1.390	1.145	110.6
0	75	45	Persian	161.25	3.325	1.390	1.127	111
0	75	60	Persian	163.75	2.925	1.397	1.105	94.9
0	40	15	Persian	136.25	3.400	1.197	1.172	97
0	40	30	Persian	134.50	3.475	1.202	1.172	98
0	40	45	Persian	133.25	3.125	1.202	1.130	98.1
0	40	60	Persian	124.50	2.900	1.202	1.067	149.7
50	100	15	Persian	513.50	6.125	2.892	3.095	149.7
50	100	30	Persian	511.00	5.975	2.892	3.115	149.9
50	100	45	Persian	503.75	5.550	2.955	3.120	150.1
50	100	60	Persian	499.50	5.375	2.897	3.437	120.2
50	75	15	Persian	297.25	5.325	1.825	2.137	120.6
50	75	30	Persian	291.25	5.225	1.845	2.125	120.8
50	75	45	Persian	287.00	4.550	1.852	2.117	120.8
50	75	60	Persian	282.75	4.500	1.852	3.100	120.8
50	40	15	Persian	254.50	4.325	1.592	2.110	124.9
50	40	30	Persian	248.75	3.900	1.595	2.090	125.7
50	40	45	Persian	245.00	3.800	1.595	2.057	126
50	40	60	Persian	239.00	3.525	1.595	2.147	126.1
100	100	15	Persian	774.00	12.625	8.375	3.642	174.9
100	100	30	Persian	772.00	12.975	8.620	3.597	178.5
100	100	45	Persian	768.75	12.875	8.720	3.607	179.3
100	100	60	Persian	766.50	13.00	8.880	3.600	179.6
100	75	15	Persian	622.25	9.700	7.362	3.487	175.5
100	75	30	Persian	619.25	10.075	7.807	3.452	178.5
100	75	45	Persian	626.25	10.175	7.895	3.432	180.1
100	75	60	Persian	625.04	10.325	8.115	3.425	189.5
100	40	15	Persian	540.25	8.675	5.325	3.437	165.5
100	40	30	Persian	539.75	8.525	5.962	3.307	166.3
100	40	45	Persian	535.75	8.475	5.980	3.310	166.6
100	40	60	Persian	538.50	8.325	6.212	3.305	166.8
150	100	15	Persian	511.00	7.625	3.192	3.262	156.2
150	100	30	Persian	508.25	7.400	3.295	3.340	156.5
150	100	45	Persian	503.75	7.200	3.360	3.232	156.8
150	100	60	Persian	499.50	7.075	3.370	3.265	157
150	75	15	Persian	459.00	5.225	2.650	2.447	140.2
150	75	30	Persian	453.50	5.150	2.710	2.427	140.2
150	75	45	Persian	449.75	4.525	2.732	2.425	140.6
150	75	60	Persian	446.50	4.425	2.750	2.402	140.6
150	40	15	Persian	404.25	4.400	2.120	2.280	135.2
150	40	30	Persian	397.50	3.975	2.340	2.285	135.8
150	40	45	Persian	388.00	3.675	2.420	2.240	136
150	40	60	Persian	382.00	3.350	2.475	2.185	136.2
0	100	15	Mexican	1024.50	8.150	3.240	2.577	345.5
0	100	30	Mexican	1017.50	8.275	3.660	2.590	347.6
0	100	45	Mexican	1014.75	8.425	3.830	2.590	348.2
0	100	60	Mexican	1009.25	8.575	4.297	1.265	348.6
0	75	15	Mexican	458.25	2.375	1.062	1.057	191.8
0	75	30	Mexican	455.50	2.125	1.072	1.032	192.1
0	75	45	Mexican	443.75	2.050	1.072	1.017	192.4
0	75	60	Mexican	438.50	1.825	1.072	1.035	192.5
0	40	15	Mexican	370.00	1.650	0.850	1.080	179.1
0	40	30	Mexican	362.50	1.300	0.852	1.037	178.7
0	40	45	Mexican	351.50	1.150	0.855	1.030	177
0	40	60	Mexican	349.25	1.125	0.855	1.025	177
50	100	15	Mexican	826.00	3.750	1.992	1.822	330.1
50	100	30	Mexican	820.50	3.500	2.025	1.880	330.4
50	100	45	Mexican	814.50	3.425	2.042	1.880	330.5
50	100	60	Mexican	811.75	3.150	2.052	1.880	330.5
50	75	15	Mexican	485.00	2.775	1.347	1.233	220.6
50	75	30	Mexican	476.25	2.600	1.375	1.217	220.8
50	75	45	Mexican	470.00	2.550	1.387	1.212	221.3
50	75	60	Mexican	465.25	2.375	1.450	1.135	221.4
50	40	15	Mexican	482.50	2.700	1.192	1.235	215.6
50	40	30	Mexican	461.50	2.225	1.197	1.217	216.2
50	40	45	Mexican	453.00	2.075	1.200	1.225	216.5
50	40	60	Mexican	445.50	1.550	1.202	1.250	216.7
100	100	15	Mexican	1063.25	8.375	6.135	2.855	344.5
100	100	30	Mexican	105.50	8.750	6.410	2.845	347.2
100	100	45	Mexican	1059.25	8.900	6.680	2.810	347.7
100	100	60	Mexican	1061.00	9.025	6.785	2.795	348.4
100	75	15	Mexican	1022.00	5.700	5.172	2.640	345.6
100	75	30	Mexican	1023.50	5.825	5.580	2.650	347.4
100	75	45	Mexican	1019.25	5.975	5.755	2.630	348.1
100	75	60	Mexican	1013.75	6.100	5.955	2.602	348.7
100	40	15	Mexican	877.50	4.500	3.137	2.385	339
100	40	30	Mexican	875.25	4.425	3.650	2.360	340.6
100	40	45	Mexican	874.25	4.325	3.815	2.275	341.1
100	40	60	Mexican	827.50	4.175	4.377	2.260	341.8
150	100	15	Mexican	827.50	4.300	2.390	2.120	332.9
150	100	30	Mexican	823.00	4.100	2.375	2.110	333.3
150	100	45	Mexican	820.00	3.975	2.420	2.072	333.4
150	100	60	Mexican	815.00	3.825	2.422	2.070	333.4
150	75	15	Mexican	770.50	3.025	1.692	1.655	320.6
150	75	30	Mexican	763.50	2.625	1.715	1.635	321.2
150	75	45	Mexican	745.00	2.550	1.732	1.617	321.2
150	75	60	Mexican	757.50	2.100	1.755	1.612	321.2
150	40	15	Mexican	626.75	2.400	1.132	1.440	316
150	40	30	Mexican	617.25	1.925	1.285	1.405	316.9
150	40	45	Mexican	602.50	1.725	1.390	1.395	317.2
150	40	60	Mexican	593.00	1.625	1.447	1.395	317.5

### Comparison of MLP, RBF, and GRNN models

**Fig 1:**
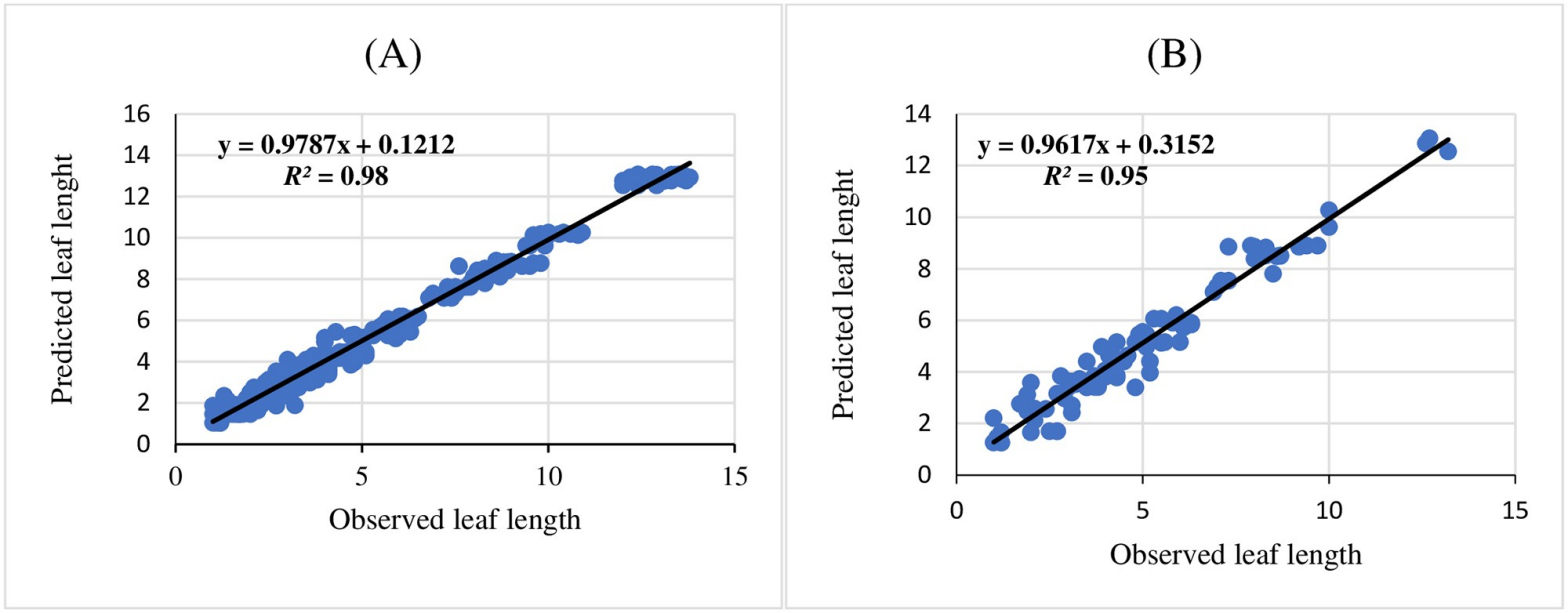
Scatter plot of observed vs. predicted results of leaf length obtained by GRNN model including (A) training set and (B) testing set.

**Fig 2:**
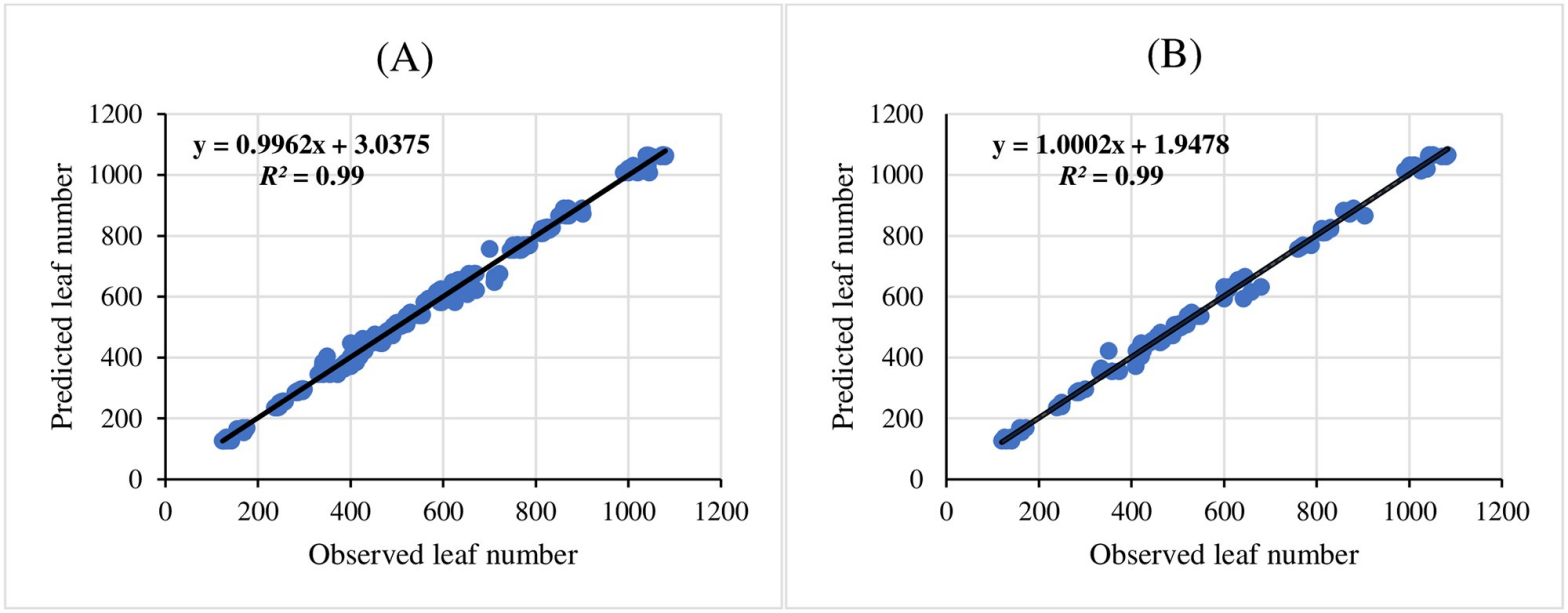
Scatter plot of observed vs. predicted results of number of leaves/plants obtained by GRNN model including (A) training set and (B) testing set.

**Fig 3:**
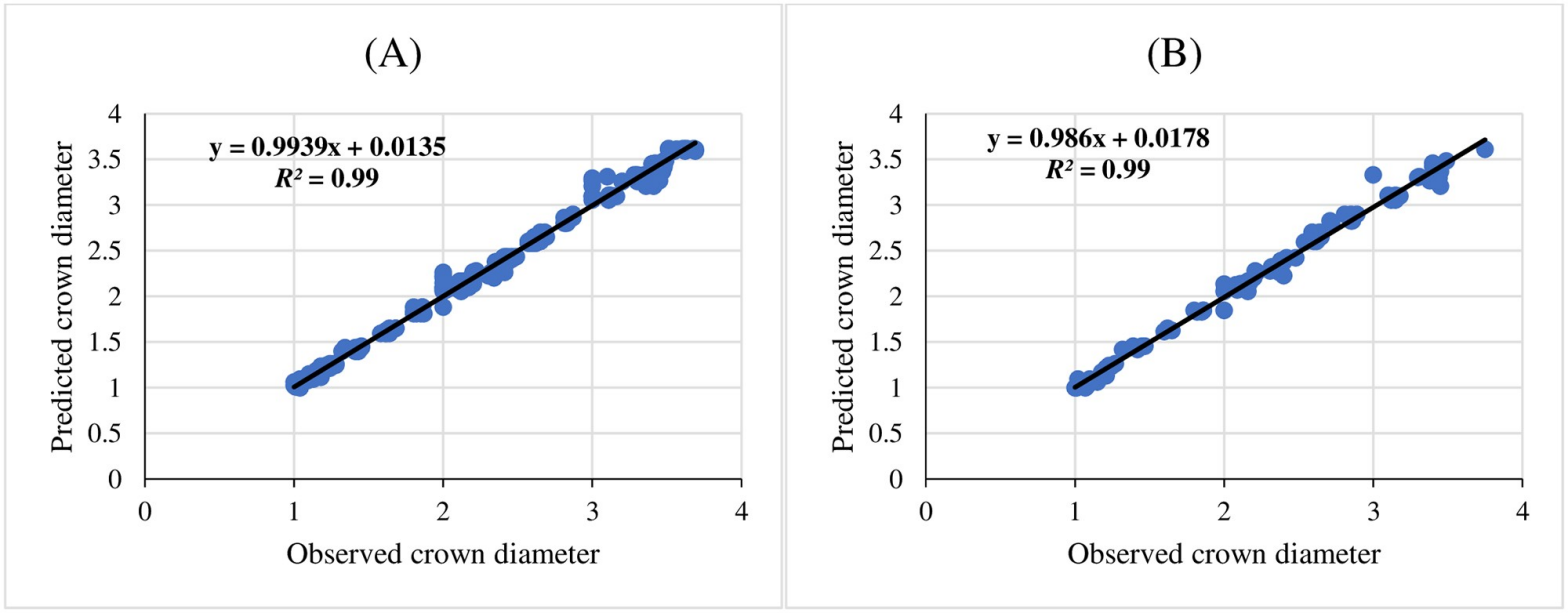
Scatter plot of observed vs. predicted results of crown diameter obtained by GRNN model including (A) training set and (B) testing set.

**Fig 4:**
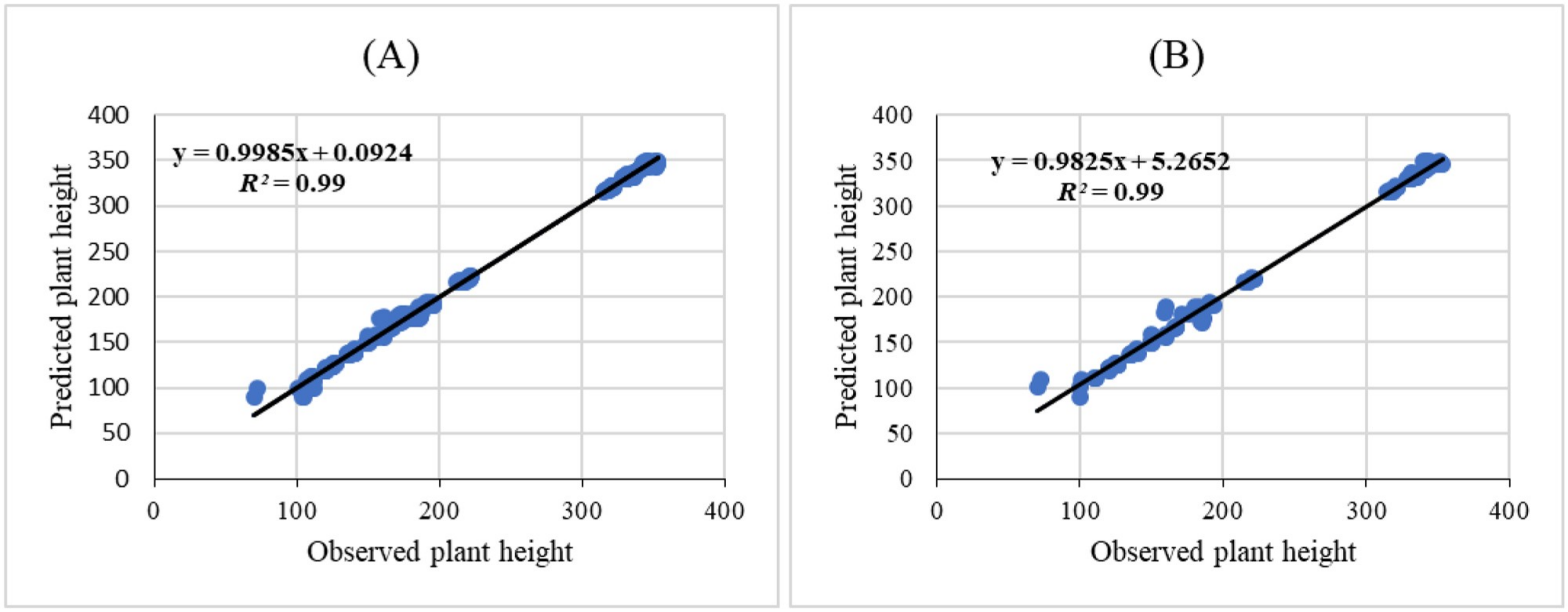
Scatter plot of observed vs. predicted results of plant height obtained by GRNN model including (A) training set and (B) testing set.

**Fig 5:**
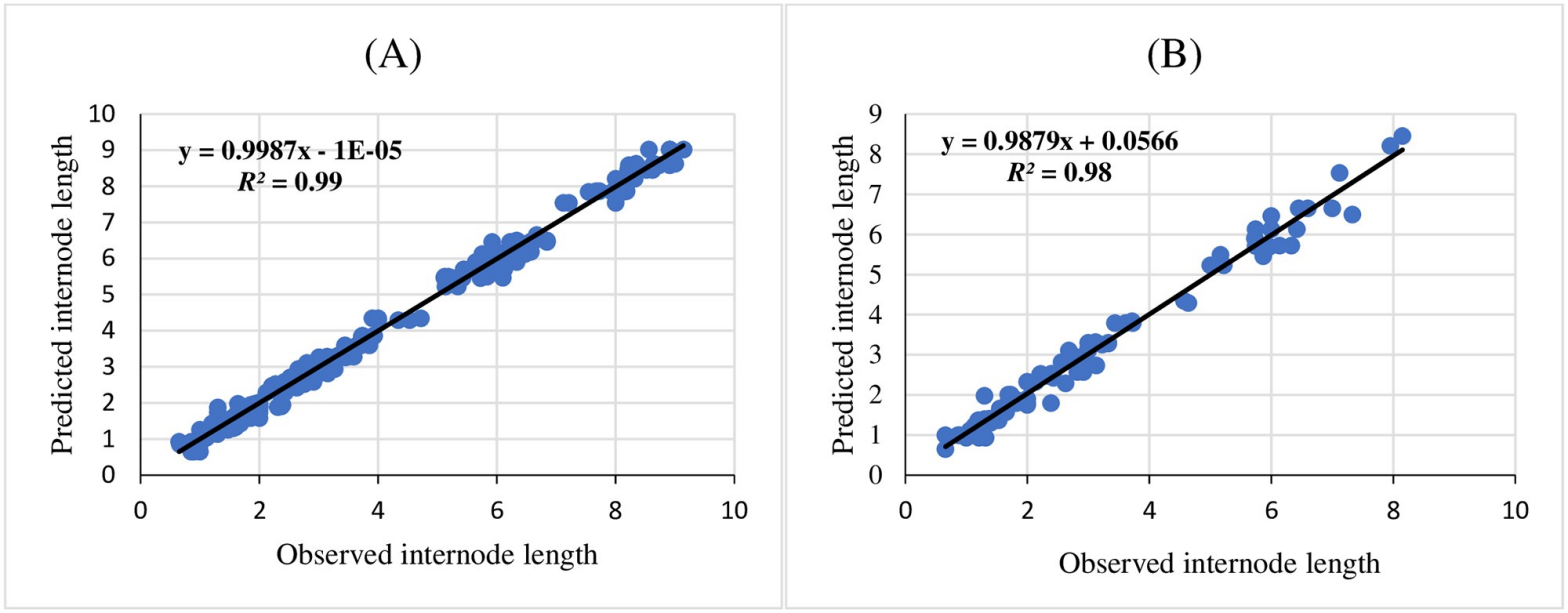
Scatter plot of observed vs. predicted results of internode length obtained by GRNN model including (A) training set and (B) testing set.

Three ANNs including MLP, RBF, and GRNN were used for modeling and predicting the effect of melatonin on morphological responses of citrus to drought stress. R^2^, RMSE, and MBE of each developed model were presented in Table 2. High significant R^2^ value and low RMSE and MBE values displayed the model capability. The results showed that GRNN and RBF had better prediction accuracy than MLP for all studied parameters. Also, comparing the results of GRNN and RBF (Table 2) revealed that GRNN is more accurate than RBF in all studied parameters. The regression lines (Figs 1–5) demonstrated that a good fit correlation between the forecasted and observed data of all studied parameters for both the training and testing sets in GRNN models.

**Table 2:** Comparison statistics of different ANNs including MLP, GRNN, and RBFNN for modeling and predicting the effect of melatonin on morphological responses of citrus to drought stress.

Model	Measured parameter		Training			Testing	
		R^2^	RMSE	MBE	R^2^	RMSE	MBE
MLP	leaf length	0.89	1.02	-0.93	0.87	1.34	0.86
	number of leaves/plants	0.91	29.61	6.53	0.89	36.57	9.11
	crown diameter	0.94	0.68	-0.08	0.88	0.66	1.05
	plant height	0.89	9.09	1.08	0.88	11.49	-1.87
	internode length	0.91	1.23	-0.08	0.91	1.23	-1.09
GRNN	leaf length	0.98	0.41	0.003	0.95	0.60	0.13
	number of leaves/plants	0.99	16.07	0.82	0.99	17.86	2.07
	crown diameter	0.99	0.06	-0.0001	0.99	0.07	-0.01
	plant height	0.99	4.11	-0.23	0.99	7.27	1.35
	internode length	0.99	0.19	-0.004	0.98	0.25	0.02
RBF	leaf length	0.94	0.68	0.06	0.91	1.02	-0.53
	number of leaves/plants	0.98	18.23	1.36	0.96	26.32	6.12
	crown diameter	0.99	0.12	0.02	0.98	0.19	-0.57
	plant height	0.96	6.03	-0.76	0.94	9.43	-1.48
	internode length	0.98	0.95	-0.07	0.95	0.68	0.94

### Optimizing morphological parameters through NSGA-II

NSGA-II was linked to the GRNN in order to determine the optimal level of melatonin concentrations, days after applying treatments, citrus species, and level of drought stress for obtaining the best morphological responses to drought stress. The results of the optimization process were presented in Table 3. Based on optimization results, applying 89.63 melatonin on Mexican lime under 94.65 FC after 53.69 days resulted in 12.35 cm leaf length, 1063.21 leaves/plant, 3.41 cm crown diameter, 324.63 cm plant height, and 8.77 cm internode length.

**Table 3:** The results of GRNN-NSGA-II to find the optimal level of melatonin concentrations, days after applying treatments, citrus species, and level of drought stress for obtaining the best morphological responses to drought stress.

input variable				leaf length	number of leaves/plants	crown diameter	plant height	internode length
melatonin concentrations	days after applying treatments	citrus species	level of drought stress					
89.63	53.69	Mexican Lime	94.65	12.35	1063.21	3.41	324.63	8.77

### Sensitivity analysis of the models

The results of sensitivity analysis were summarized in Table 4. Based on sensitivity analysis, leaf length and number of leaves/plants were more sensitive to melatonin concentration, followed by level of drought stress, days after applying treatments, and species. Also, as can be seen in Table 4, melatonin concentration was the most important factor for crown diameter, followed by species, days after applying treatments, and level of drought stress, respectively. Also, plant height was more sensitive to species, followed by melatonin concentration, days after applying treatments, and level of drought stress. Moreover, melatonin concentration was the most important factor for internode length, followed by days after applying treatments, species, and level of drought stress (Table 4).

**Table 4:** The results of sensitivity analysis to find the importance of each input in morphological responses to drought stress.

Output	Item	Melatonin concentrations	Days after applying treatments	Citrus species	Level of drought stress
leaf length	VSR	3.36	2.76	1.97	2.94
	Rank	1	3	4	2
number of leaves/plants	VSR	1.94	1.28	1.09	1.75
	Rank	1	3	4	2
crown diameter	VSR	2.09	1.82	1.95	1.65
	Rank	1	3	2	4
plant height	VSR	2.31	1.73	2.65	1.66
	Rank	2	3	1	4
internode length	VSR	2.03	1.74	1.36	1.15
	Rank	1	2	3	4

### Validation experiment

According to the validation experiment, the differences between experimental validation data and predicted data via GRNN-NSGA-II were not significant. Therefore, it can be concluded that GRNN-NSGA-II with better performance than RBF or MLP can be employed for accurately predicting and optimizing the effects of melatonin on morphological responses under drought stress.

## Discussion

Drought can be considered as one of the most common abiotic stresses that restricts plant growth and development as well as productivity through changing different morphological, biochemical, physiological, and molecular pathways [50]. Continuous drought stress leads to a water shortage inside the different plant cells, tissues, and organs. Moreover, the effects of drought stress are usually first observed in the leaf tissues which gradually fold and curl, and then, are seen in whole plant growth and development [51]. By losing water content, the leaves under drought stress have initiated to roll up and turn yellow and necrosis. Also, drought stress resulted in the decline in plant height which might be due to the decreases in cell turgor, cell growth, cell elongation, and cell volume [52]. Previous studies showed that drought stress plays a pivotal role in decreasing water contents, inhibiting cell expansion and division, increasing osmotic stress, and, generally declining plant growth [53]. In the current study, drought stress led to inhibition of citrus seedlings growth. However, the results of the current study indicated that the negative impacts of drought stress in both citrus genotypes could be mitigated by the application of melatonin through improving morphological responses. Also, our results elucidated that the melatonin-treated plants in comparison with untreated plants exhibited larger plant height, leaf number and length, crown diameter and internode length with better tolerance potential under the water scarcity due to the assimilates of water and osmolytes to growing cells and tissues. A high growth of leaf (length / number) during drought stress might be due to the improved stomatal conductance related to the foliar-sprayed melatonin. Therefore, leaf growth via cell proliferation and expansion may be influenced by exogenous application of melatonin. Indeed, the duration and rate of cell division, as essential parameters of cell proliferation, play an important role in the leaf size [54]. In line with our results, previous studies revealed that the exogenous application of melatonin can improve morphological responses under drought stress in different plants such as tobacco [12], *Camellia sinensis* [13], cucumber [14], soybean [15], alfalfa [16], rice [17], maize [18], cotton [19], grape [20], and kiwi [21]. It seems that melatonin as a novel plant growth regulator, independently of auxin signaling, plays a pivotal role in shoot and root growth and development [10, 11]. Although melatonin at a lower level improves growth and development, a higher level of this plant growth regulator has an inhibitory function; therefore, melatonin can be considered as a dose-dependent growth regulator [55].

Morphological changes to drought stress are a multivariable process that is influenced by several factors. Also, morphological responses to drought stress are a highly complex and nonlinear process. Therefore, there is a serious need to employ robust nonlinear computational methods for analyzing plant biological processes. The efficiency of a good statistical approach depends on the neat understanding of the variable structure, experimental design, and using the appropriate model [56]. One of the most important primary requirements to identify suitable statistical approaches is comprehending the type of data [57, 58]. Variables can be clustered into two groups including quantitative (continuous and discrete) and qualitative (ordinal and nominal). Names with two or more classes without a hierarchical order are categorized as nominal variables, while ordinal data have distinct order (level X is more intense than level Y) [57, 59]. Counts that include integers are classified as discrete data, while measurements along a continuum, which could be included smaller fractions are categorized as continuous variables [60]. Plant biological data can be categorized as ordinal (plant quality rated as weak, moderate, and good), nominal (type of morphological responses such as normal and necrosis), continuous (height of shoots or roots), and discrete (number of leaf). Traditional linear methods such as regression and ANOVA must be just applied with continuous variables that demonstrate a linear relationship between the explanatory and dependent variables [23, 61, 62]. Hence, the conventional computational approaches are not appropriate for analyzing plant biological processes [57]. Recently, different machine learning algorithms have been successfully utilized to predict and optimize various plant physiological processes. Several studies [24, 58, 63] used MLP to predict various plant biological processes. However, they only applied the MLP model and did not compare this well-known algorithm with other models. In the current study, different ANNs including GRNN, RBF, and MLP, for the first time, were used to develop a suitable model for prediction morphological responses to various concentrations of melatonin under different levels of drought stress and compare their prediction accuracy. According to our results, RBF and GRNN had more accuracy than MLP for modeling and predicting the system. Also, NSGA-II was linked to GRNN as the most suitable model for the optimization process. Based on our results, GRNN-NSGA-II can be considered as an efficient computational methodology for modeling and predicting morphological responses in the field of stress physiology. Also, according to the current results, the developed model presented here would allow more accurate estimations without the requirements for the availability of all data.

## Conclusion

In the current study, different ANNs including GRNN, MLP, and RBF along with NSGA-II were used for studying and predicting the morphological responses of citrus under drought stress for the first time. The great accordance between the predicted and observed data support the high efficiency of ANNs. Overall, ANNs effectively model complex input-output patterns and show a supreme ability for studying and predicting different plant morphological, physiological, and biochemical responses to abiotic stresses. Therefore, ANN-NSGA-II can be employed as a new computational strategy in the field of stress physiology. While ANNs usually have good performance in prediction, they are limited to explicitly illustrate the effects of explanatory variables on the response variable, due to their “black box” feature. Therefore, it would be suggested to compare ANNs with other machine-learning methods (e.g., Random Forest, Gradient Boosting, support vector machine), to allow a more thorough appreciation of the relative potential of ANNs applied to the presented problem.
